# Supplementary material for: Quantitative Metabolomics of Tissue, Perfusate, and Bile from Rat Livers Subjected to Normothermic Machine Perfusion
Source: Biomedicines. 2022 Feb 24;10(3):538. doi: 10.3390/biomedicines10030538 (PMC8945564; doi:10.3390/biomedicines10030538)

Supplementary Material

NMR spectroscopy-based metabolomic profile of tissue, perfusate, and bile from rat livers subjected to normothermic machine perfusion

Caterina Lonati, Daniele Dondossola, Laimdota Zizmare, Michele Battistin, Leonie Wüst, Luigi Vivona, Margherita Carbonaro, Alberto Zanella, Stefano Gatti, Andrea Schlegel and Christoph Trautwein

Supplementary material and methods ***2***

Supplementary Figure 2. Schematic representation of the ex-situ normothermic machine perfusion system. **3**

Supplementary Table 1. Perfusion fluid composition **4**

Supplementary results ***5***

Supplementary Table 2. Baseline perfusate composition **5**

Supplementary Figure 3. Oxygen delivery/consumption during NMP. **6**

Supplementary Figure 4. Heatmap representing the metabolite concentrations in liver tissue homogenates. **8**

Supplementary Figure 5. Heatmap representing the metabolite concentrations in the perfusate samples. **9**

Supplementary Figure 6. Heatmap representing the metabolite concentrations in bile samples. **10**

Supplementary Figure 7. Averaged heatmaps representing differentially expressed and statistically significant metabolites. **11**

Supplementary material and methods

Supplementary Figure S1. Schematic representation of the experimental workflow.
All groups received a surgery, further SCS, h-NMP and OxC-NMP had in-situ perfusion and SCS for 30 minutes. Finally, h-NMP and OxC-NMP groups as the name suggests had the NMP.

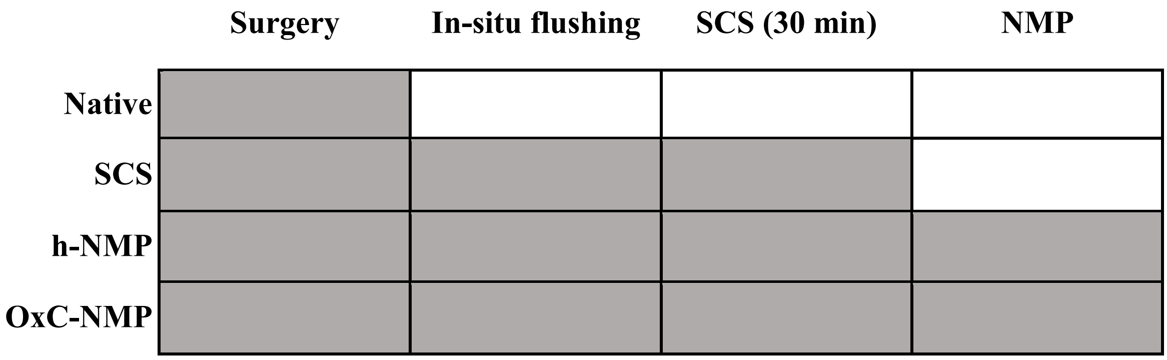

Supplementary Figure S2. Schematic representation of the ex-situ normothermic machine perfusion system.

The system was derived from an isolated lung perfusion system (Hugo Sachs Elektronik, Harvard Apparatus, March-Hugstetten, Germany) with disposable PVC tubes and membrane oxygenator (Micro-1 rat oxygenator, Kewei Rising Medical, Shenzhen, China). The perfusate flow was generated by a peristaltic pump and the membrane oxygenator received a gas mixture of 95% oxygen and 5% carbon dioxide (CO2). The liver, connected to the circuit using the portal vein cannula, was placed on a double layer of Parafilm® (Merck, KGaA, Darmastd, Germany), previously modelled and perforated to allow the drainage of the perfusate to the reservoir. Recirculating water bath was set to maintain graft temperature at 37°C that was measured with a custom-made thermocouple. Pressures along the circuit were monitored downstream the peristaltic pump and upstream the portal cannula.

A: Water Jacked reservoir, B: Peristaltic Pump, C: Membrane oxygenator, D: Heating Coil/bubble trap.

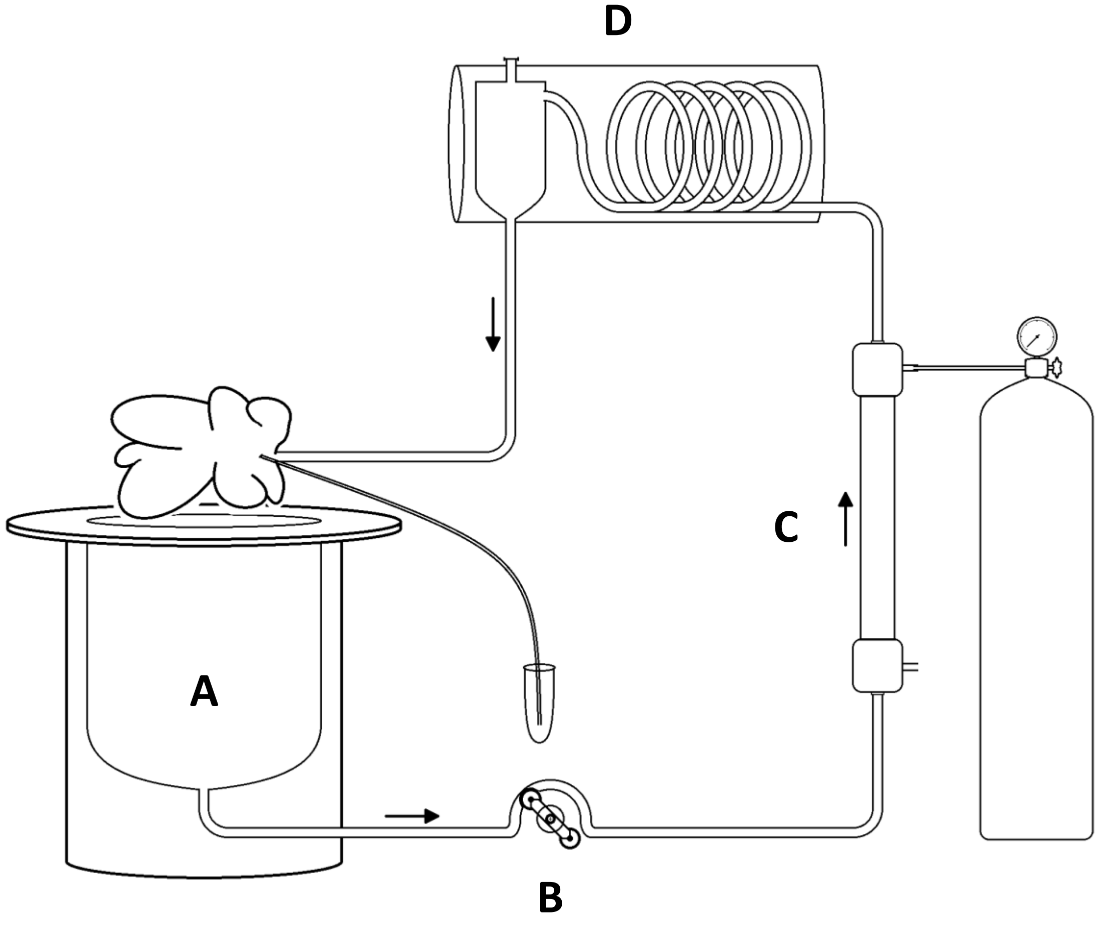

Supplementary Table S1. Perfusion fluid composition

	h-NMP		OxC-NMP	
	**mL**	**Concentration**	**mL**	**Concentration**
**DMEM**	76		37.5	
**Oxyglobin® (13 g/dL hemoglobin)**	0	0	38.5	5 g/dL
**Human Albumin (20 %)**	20	4%	20	4%
**Pen-Strep (10000 U/mL – 10 mg/mL)**	1	100 U/mL – 0.1 mg/mL	1	100 U/mL – 0.1 mg/mL
**L-Glutamine (200 mM)**	1	0.292 g/L	1	0.292 g/L
**Human Insulin (100 UI/mL)**	2	2 U/mL	2	2 U/mL
**Total Volume**	100		100	

Supplementary results

Supplementary Table S2. Baseline perfusate composition

	h-NMP (n=5)	OxC-NMP (n=5)	P value
**pH**	7.38 ± 0.09	7.16 ± 0.27	0.118
**Hb, g/dL**	-	5.2 ± 0.2	-
**Htc, %**	-	16 ± 1	-
**Gluc, g/dL**	80.00 ± 0.50	39.00 ± 1.00	<0.001
**Lac, mmol/L**	-	4.2 ± 0.2	-
**K^+, mmol/L**	4.1 ± 0.1	3.6 ± 0.1	<0.001
**Na^+, mmol/L**	149 ± 1	148 ± 2	0.166
**Ca^{2+}, mmol/L**	0.72 ± 0.28	0.85 ± 0.10	0.429
**Cl^-, mmol/L**	121 ± 2	113 ± 1	<0.001
**HCO_3^-, mmol/L**	23 ± 2	13 ± 1	<0.001

Supplementary Figure S3. Oxygen delivery/consumption during NMP.

Two-way repeated measures ANOVA; Tukey's post hoc test. DO_2: $p<0.001$; VO_2: $p<0.05$ at 5 min.

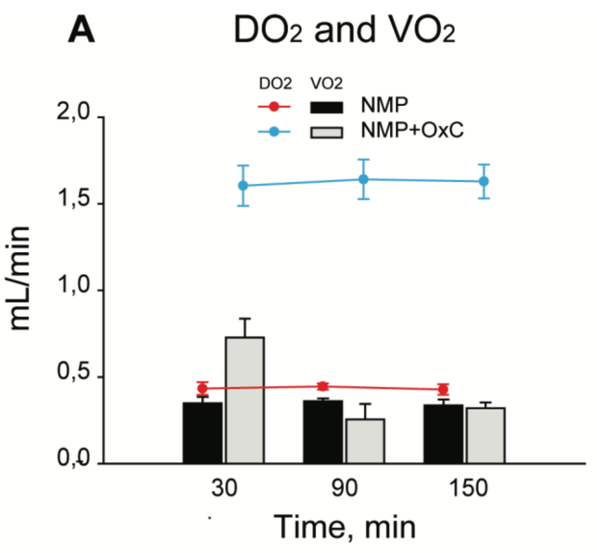

Supplementary Figure S4. Heatmap representing the metabolite concentrations in liver tissue homogenates.

Abbreviations: SCS – static cold storage, NMP – normothermic machine perfusion, h-NMP – hypoxic NMP, OxC-NMP – oxygen carrier NMP, UMP – uracil monophosphate, IMP – inosine monophosphate, GSSG – glutathione disulfide, UDP – uracil diphosphate.

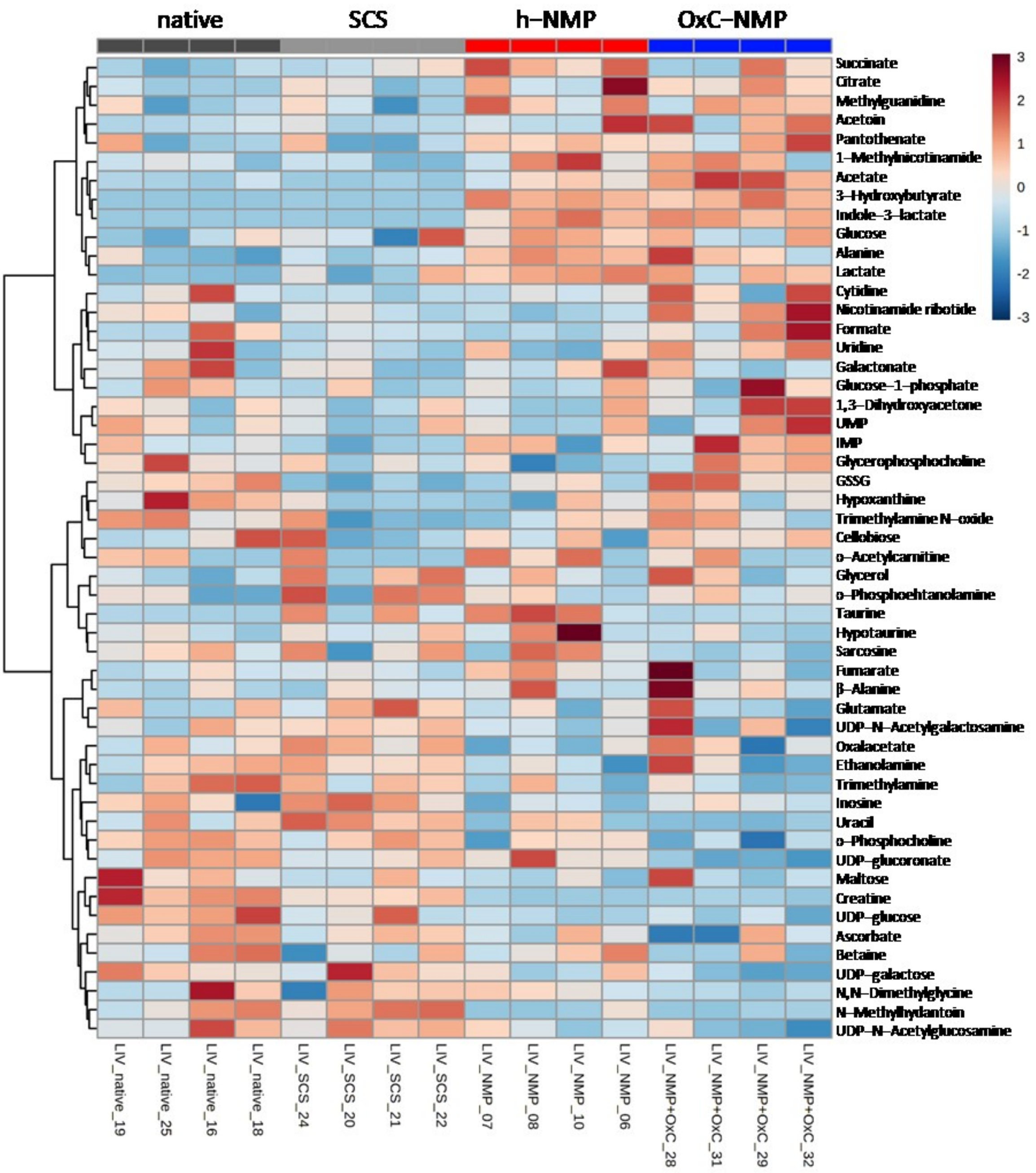

Supplementary Figure S5. Heatmap representing the metabolite concentrations in the perfusate samples.

Abbreviations: SCS – static cold storage, NMP – normothermic machine perfusion, h-NMP – hypoxic NMP, OxC-NMP – oxygen carrier NMP.

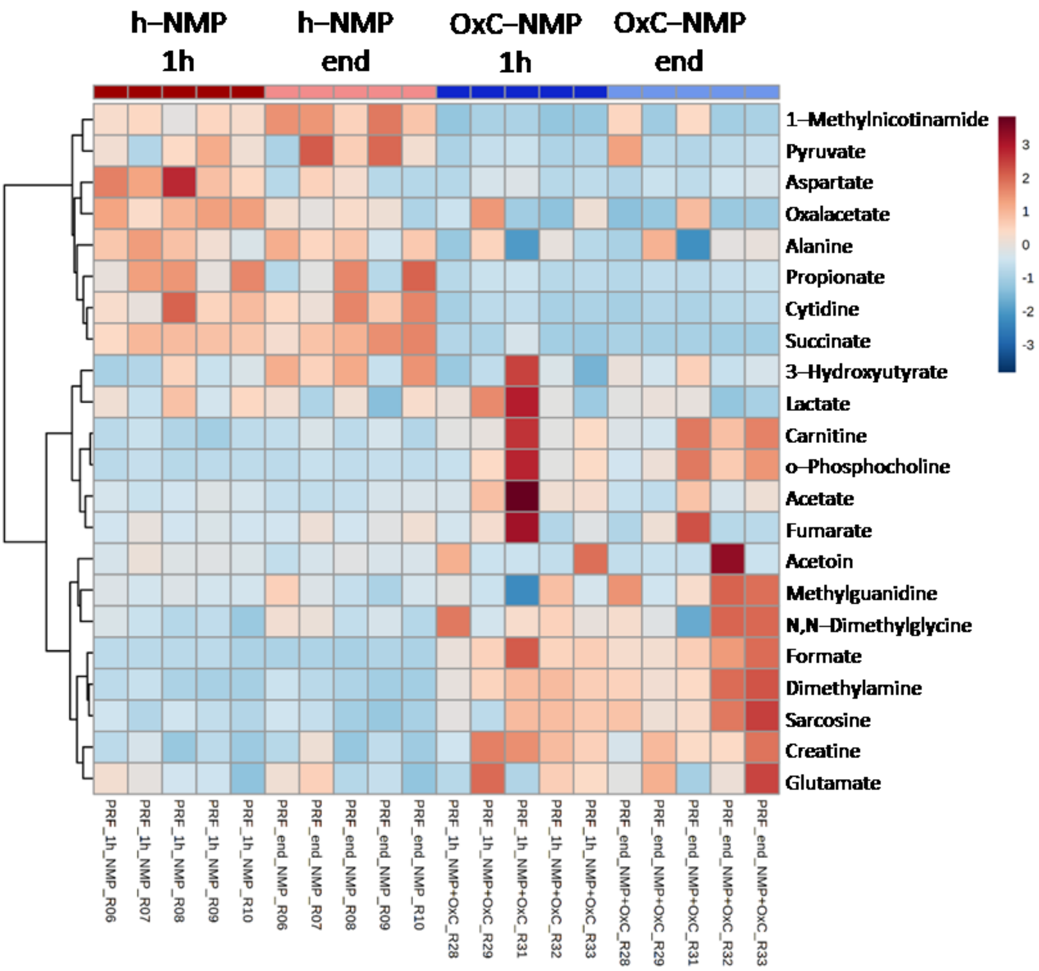

Supplementary Figure S6. Heatmap representing the metabolite concentrations in bile samples. Abbreviations: SCS – static cold storage, NMP – normothermic machine perfusion, h-NMP – hypoxic NMP, OxC-NMP – oxygen carrier NMP, c – carbon, GSSG – glutathione disulfide.

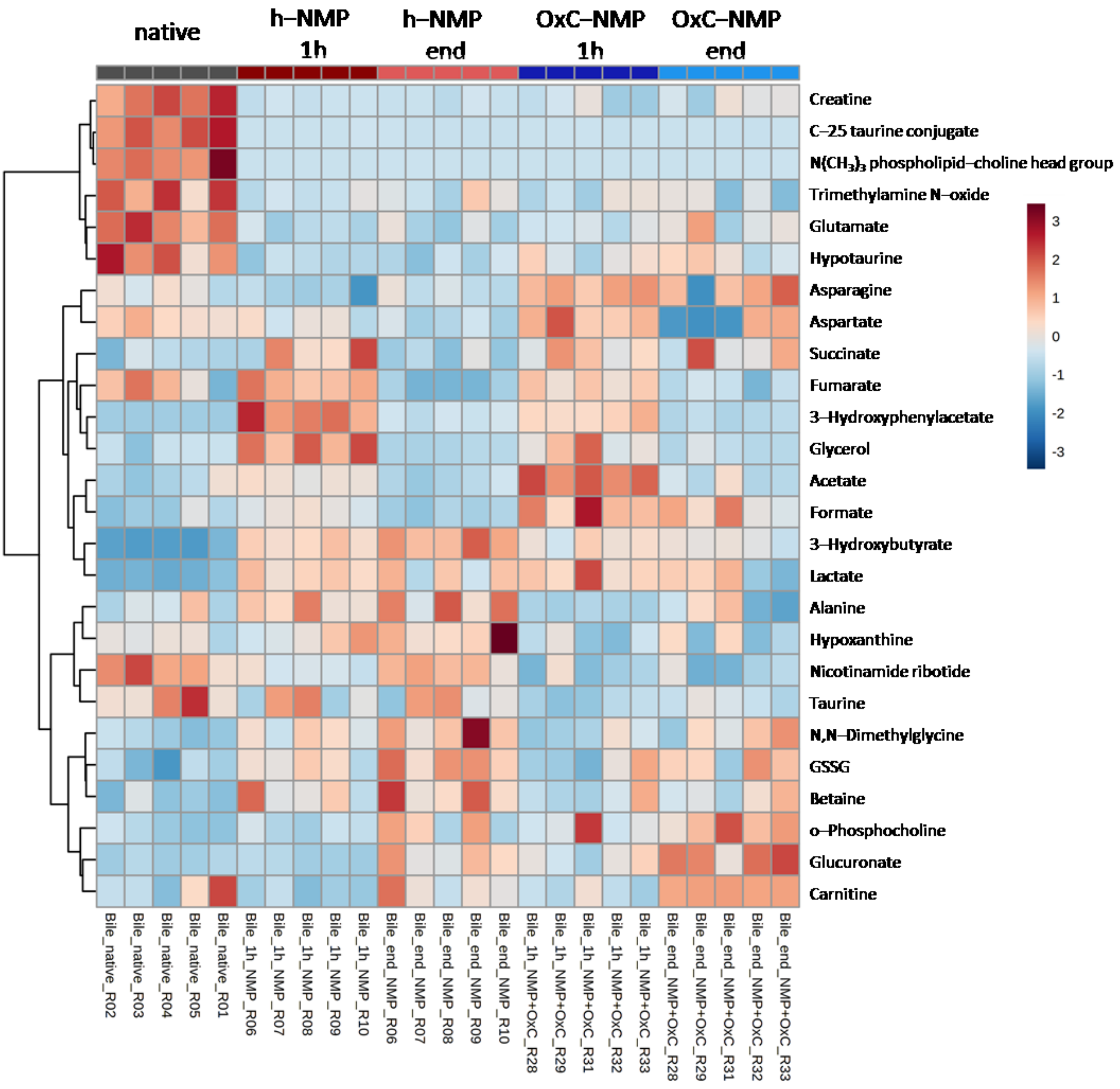

Supplementary Figure S7. Averaged heatmaps representing differentially expressed and statistically significant metabolites.

(a) liver biopsies; (b) perfusate samples. One-way ANOVA, p<0.05.

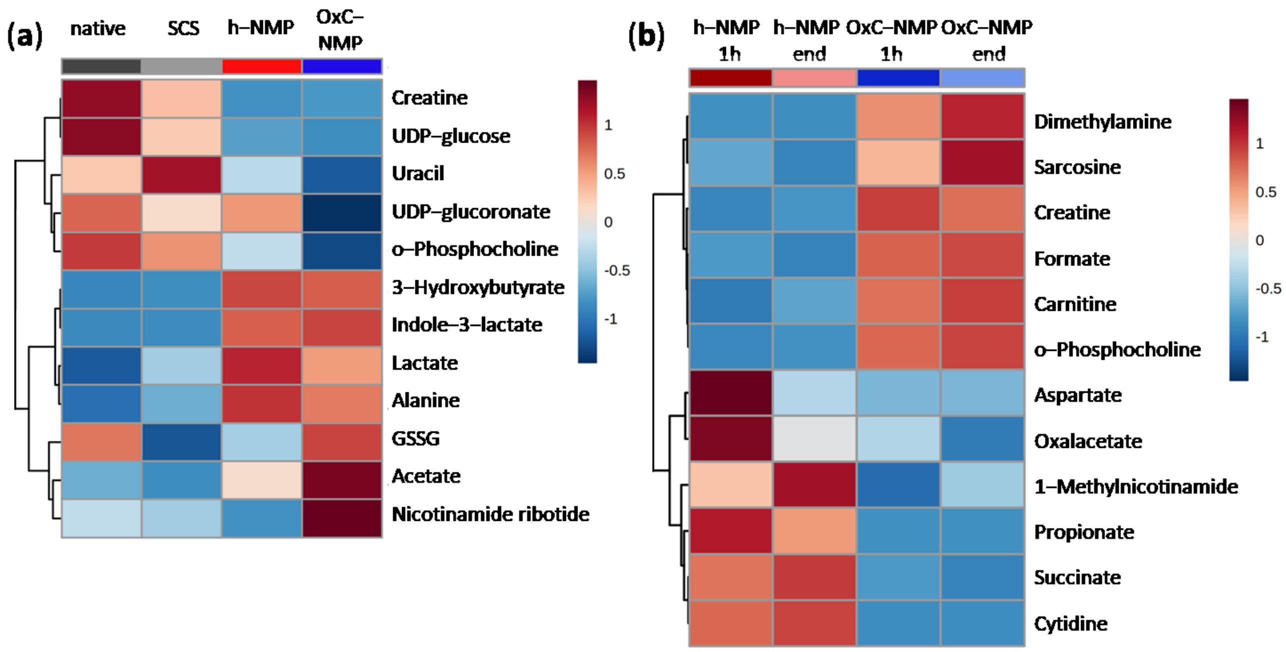

Supplement: Supplementary file 1 [file biomedicines-10-00538-s001.zip › biomedicines-1598961-supplementary.pdf]
